# Hospitals of To-Day

**Published:** 1911-04-29

**Authors:** 


### HOSPITALS OF TO-DAY.

### III.—THE WEST LONDON HOSPITAL AND ITS FUTURE.

The West London Hospital is an institution which has long prepared a scheme of development so that the populous suburbs still springing up round Hammersmith, with the number of cases which they bring to the out-patient department, have not taken it unawares. Several years ago a scheme of extension was laid before King Edward's Hospital Fund which, if adopted fully, would have completed the buildings in accordance with previously accepted plans. The cost of this scheme was expected to be £63,000, and it included three matters of urgency for which £27,000 was required. The King's Fund, however, was compelled to advise the postponement of all extension till the debt of £12,000 which has persistently hampered the hospital for a lamentably long time should be paid. It is worth the notice of all the institution's supporters that this sum has shown only a small tendency to increase, and that the hospital otherwise has managed to pay its way, and has been hindered from healthy expansion only by the incubus of this tenacious deficit.

The result has been that the West London Hospital to-day finds itself without a nurses' home in the modern sense at all, and is unable to guarantee to each member of the nursing staff that room to herself which has now been established as a necessity in every twentieth-century hospital. This extension to the nurses' quarters has been felt by the Board as so imperative that it has been placed first on the list of urgent requirements. A nurses' home on up-to-date lines has been planned at a cost of £9,000, but the payment of the debt must precede all building operations. At the same time, moreover, the pathological department of the hospital is in need of modernisation. Neither the laboratories nor the mortuary, as they exist to-day, satisfy the standard with which the West London Hospital authorities are anxious to conform. There is no need to emphasise the fact that in the last few years the importance attached to bacteriology has grown so much as to make new pathological departments one of the commonest demands of hospitals to-day. The West London Hospital, owing to its debt, is unable to do all that it might under more modernised conditions.

We now come to the most expensive of the three improvements that are pressingly needed, namely, a new administrative block, which would complete the frontage to Hammersmith Road, at a cost of £14,000. The out-patient department, as has been hinted, is too small for the number of cases treated. Over 43,000 were received last year—that is to say, an increase of 4,000 on the number received only twelve months earlier. A system of inquiries of course obtains, but the West London Hospital has not adopted official almoners. As it is essentially a consultative department, the need for them is less here than elsewhere. The growth in the number of out-patient cases makes the need for extended quarters sufficiently obvious, and no one will be surprised to hear that the consulting rooms and waiting hall, which, by the way, is well lighted and on the ground floor, are cramped and exiguous. In connection with the proposed extensions it is most important to add that the West London Hospital is fortunate in its site, which lies four-square to Hammersmith Road, and which includes property at the back. That, being owned by the institution, can be pulled down or adapted as required. The problem of extension here is not complicated, as in the case of the Chelsea Hospital for Women, for instance, by inherent difficulties of site.

Such is the present position. In other words, it will be seen that once the debt of £12,000 be removed, progressive developments could go actively forward. For some reason or other, perhaps the scattered area which it serves, the hospital has not received all the support it deserves from West London. At the present moment, however, thanks partly to Hammersmith and partly to the hospital's own initiative, there seems a prospect of the debt being lightened, if not extinguished, by the end of the year. King Edward's Hospital Fund recently set the locality a splendid example by nearly doubling its previous contribution. Hammersmith, in the person of its Mayor, Mr. Norman Shairp, has decided that its memorial to King Edward shall be a ward named after him at the hospital, and it is hoped that, if the energy of the promoters of this scheme are not allowed to flag, the £3,000 necessary will quickly be obtained for the purpose. There is no doubt that a still larger sum would be forthcoming if the hospital's claims were brought home to every one in this great district. The proposed house-to-house collection, therefore, should produce a handsome sum, if every house be really visited.

The hospital itself has decided to hold a Coronation Festival Dinner at the White City on June 29, when the Duke of Abercorn will preside, and great results are expected, for the hospital's claims will be brought before the public at the moment when the spending powers of London are being exercised most widely. It is most important that this function should be a success, for the debt must be paid off, and it would be a great encouragement if a balance could also be handed over to the Building Fund, which was started in 1909, and which now amounts to over £800. In conclusion, it should be pointed out that the proper housing of the nurses is dependent on the enlargement of the out-patient department, and that, therefore, any support which Hammersmith or the public generally gives to the hospital will have the double advantage of helping both schemes. That the hospital is doing what it can to improve its position appears from the appointment last autumn of a special finance committee to deal with ways and means. The financial scheme just outlined is the result of its endeavours. It is clear, then, that the hospital's future development depends very much on the strength of the support which it receives this year.

